# Identification of candidate risk gene variations by whole-genome sequence analysis of four rat strains commonly used in inflammation research

**DOI:** 10.1186/1471-2164-15-391

**Published:** 2014-05-21

**Authors:** Liselotte Bäckdahl, Diana Ekman, Maja Jagodic, Tomas Olsson, Rikard Holmdahl

**Affiliations:** Medical Inflammation Research, Department of Medical Biochemistry and Biophysics, Karolinska Institutet, Stockholm, Sweden; Center for Molecular Medicine, Department of Clinical Neuroscience, Neuroimmunology Unit, Karolinska Institutet, Stockholm, Sweden; Unit of Medical inflammatory disease, Department of Medical Biochemistry, Karolinska Institutet, Scheeles väg 2, B2 plan 4, 171 77 Stockholm, Sweden

**Keywords:** Rat genome, Whole-genome sequencing, QTL, Rheumatoid arthritis, Multiple sclerosis, Disease-associated SNVs

## Abstract

**Background:**

The DA rat strain is particularly susceptible to the induction of a number of chronic inflammatory diseases, such as models for rheumatoid arthritis and multiple sclerosis. Here we sequenced the genomes of two DA sub-strains and two disease resistant strains, E3 and PVG, previously used together with DA strains in genetically segregating crosses.

**Results:**

The data uncovers genomic variations, such as single nucleotide variations (SNVs) and copy number variations that underlie phenotypic differences between the strains. Comparisons of regional differences between the two DA sub-strains identified 8 genomic regions that discriminate between the strains that together cover 38 Mbp and harbor 302 genes. We analyzed 10 fine-mapped quantitative trait loci and our data implicate strong candidates for genetic variations that mediate their effects. For example we could identify a single SNV candidate in a regulatory region of the gene *Il21r,* which has been associated to differential expression in both rats and human MS patients. In the *APLEC* complex we identified two SNVs in a highly conserved region, which could affect the regulation of all APLEC encoded genes and explain the polygenic differential expression seen in the complex. Furthermore, the non-synonymous SNV modifying aa153 of the Ncf1 protein was confirmed as the sole causative factor.

**Conclusion:**

This complete map of genetic differences between the most commonly used rat strains in inflammation research constitutes an important reference in understanding how genetic variations contribute to the traits of importance for inflammatory diseases.

**Electronic supplementary material:**

The online version of this article (doi:10.1186/1471-2164-15-391) contains supplementary material, which is available to authorized users.

## Background

The laboratory rat is a widely used model organism for studying both basic mechanisms of physiology, and disease models of human diseases. Inbred rat strains and genetically segregating crosses are important tools in functionally proving disease mechanisms and represents a controlled setting where molecular interactions between different genetic factors as well as environmental factors can be tested [1].

There are many common characteristics between chronic inflammatory diseases like Rheumatoid Arthritis (RA) [2], and Multiple Sclerosis (MS) [3]: these diseases are both consequences of dys-regulated immune responses that has initially been triggered by a combination of genetic and environmental factors. Peripheral joints in RA and myelin sheets in the central nervous system in MS are the sites of persistent inflammation that leads to tissue destruction. The most highly associated genetic region for the majority of these diseases is the Major Histocompatibility Complex (MHC) and especially HLA II genes [4]. Despite recent progress in identifying loci that associate with complex diseases in human [5], the identification of the causative genetic polymorphisms, (both within and outside MHC), and the understanding of their pathophysiological roles, still represents a significant challenge. The complexity of chronic inflammatory diseases lies not only in intricate interactions between genes and environment, but also in elaborate genetic phenomena such as gene-gene interactions, synergistic effects and epigenetic mechanisms.

Animal models provide a means to control environmental factors and manipulate the genetic contribution. Although the disease causing polymorphisms are not likely to be identical with humans we expect the associated pathophysiologic pathways to be conserved. Using animal models could be a way to reveal their genetic complexity. In addition, the environmental exposure can be controlled and experimental manipulations are possible to perform. One other benefit of using animal models for genetic research is the possibility to selectively modify the genome e.g. producing congenic, knock-out or transgenic strains and linkage mapping.

**Figure 1:**
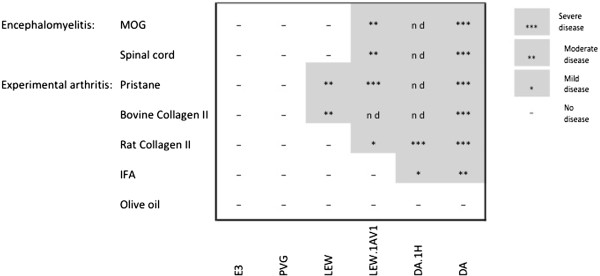
**The genomic influence on disease susceptibility.** The influence of genomic variations on the development of inflammatory disease by different genomes and MHC haplotypes. The illustration is an adaptation from [14].

DA rats are remarkably susceptible to a number of autoimmune diseases; experimental arthritis induced with collagen [6] or hydrocarbons such as mineral oil [7] or pristane oil [8], experimental autoimmune encephalomyelitis [9], experimental allergic neuritis [10], experimental allergic uveitis [11], and experimental autoimmune thyroiditis [12]. Also among other inbred rat strains, the DA rat is the most prone to develop inflammation (Figure 1). This disease susceptibility is mediated by both MHC and non-MHC genes. It is the only strain susceptible to oil-induced arthritis and other arthritis inducers provoke very severe disease in DA compared to other susceptible strains. It has been shown that DA rats from different colonies differ in disease susceptibility as well as contain distinct regions of genetic differences [13], therefore we decided to sequence 2 sub-strains of DA; DA/OlaHsd and DA/hanKini ( here abbreviated as DA/O and DA/K).

**Figure 2:**
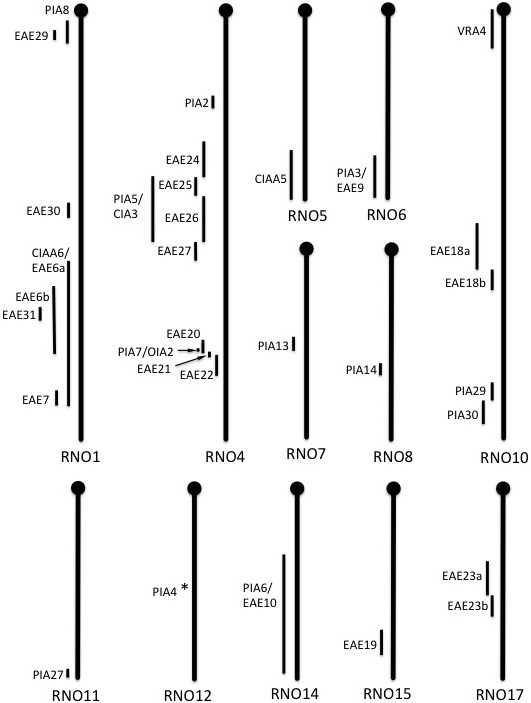
**Arthritis and EAE regulating QTLs in the DA genome.** Chromosome maps illustrating the genomic intervals of inflammatory disease regulating QTLs in the DA rat genome associated with mapping studies in DA/E3 or DA/PVG crosses and congenic strain.

Genetic linkage studies using F2 animals from an inter-cross between DA and different disease resistant strains have identified about 50 quantitative trait loci (QTLs) associated with susceptibility to inflammatory diseases. These QTLs together cover approximately 20 genomic regions in the DA rat that have been confirmed in more than one F2 inter-cross (Figure 2). The first F2 cross that used DA as the inflammation susceptible strain was a DA/K x E3 cross in Pristane induced arthritis (PIA), that identified QTLs on chromosome 6, 4, 12, and 14 [8]. Three more arthritis mapping studies and two in EAE utilizing DA/K x E3 F2 inter-crosses could verify the first four QTLs and identified new QTLs on chromosome 20 (MHC region), 10, 1, and 18 [15–19]. Dahlman et al. used a DA/K x ACI F2 cross in EAE to identify QTLs on chr 4, 7, 10, 12, 13, 15, 18, and X [20]. The QTLs on chr 4, 10, 12, 15 were later confirmed in DA/K x PVG.1AV1 advanced intercross line (AIL) [21–26]. In 1998 two arthritis QTLs were identified in LEW.1AV1x PVG.1AV1 on chr 4 and 10 that later were replicated in a DA/K x PVG.1AV1 F2 [27, 28]. In 2003 a LEW.1AV1 x PVG.1AV1 F2 cross identified two new QTLs on chromosome 1 and 17 [29] that were replicated in two DA/K x PVG.1AV1 AIL populations [30–33]. The PVG.1AV1 is a congenic strain sharing the MHC region with DA, but is still resistant or develops extremely mild disease in response to most inflammation inducers. The E3 strain is an independent inbred rat strain that is highly resistant to most inflammatory diseases but which tends to develop obesitas and has a coagulation defect [34]. Aiming to identify genomic regions that differ between disease susceptible and resistant strains, DA/O, DA/K, E3/han and PVG/1AV1.Kini were selected for sequencing.

Since the main purpose of this sequencing effort has been to create a tool to be used in all genetic studies of the inflammatory rat we have also selected 10 quantitative trait loci (QTLs) that are ~ 1 Mb in size, to dissect and report all the variations in the interval. Main emphasis is put on non-synonymous SNVs (nsSNVs), synonymous SNVs (sSNVs), SNVs in UTRs, splice-site SNVs and SNVs found in regions of high conservation between different species (PhastCons9way). The new SNVs that are identified will have to be analyzed in more detail and assessed experimentally. However, they represent an entry point for functional validation of candidate molecular genetic mechanisms underlying established QTLs.

The identified QTLs that segregate between DA and E3/PVG signify regions of regulatory importance in the development of chronic inflammatory disease. Identifying the underlying factors within these disease-regulating regions would give important clues also for the diseases in human. The complete genomic sequence of the DA strain and the disease resistant strains E3 and PVG comprise invaluable resources in the study of the polygenic nature of complex diseases such as chronic inflammatory diseases.

## Results and discussion

### Sequencing and variation calling

The four genomes DA/OlaHsd (DA/O), DA/hanKini (DA/K), E3/han (E3) and PVG/1AV1.Kini (PVG) were sequenced in three separate runs using the SOLiD™ technology. Aiming to also identify structural variations we made three Mate-pair libraries with different insert sizes; 1 kb, 2 kb, and 4 kb as well as one paired end library. The reads were then mapped to the BN (RGSC3.4) reference genome with BWA [35]. This resulted in median coverage of 18-22X (DA/O: 18X, DA/K 22X, E3 19X, PVG 19X) and enabled us to cover >99% of the BN reference sequence with at least one read for all four genomes (Additional file 1).

SNVs and short indels were identified using a strategy developed by Guryev et al [36]. To call a variant we required a minimum coverage of three reads and at least 75% of the reads had to support the non-reference allele. This predicted about 5.3 million SNVs and 0.4 million short indels for all four genomes combined.

By comparing our SNVs with the genotypes from the STAR consortium [37] we estimated the sensitivity to be about 96.8-97.8%. In addition, we sequenced 100 regions (300-500 bp in size) with capillary sequencing. Of the 178 SNVs identified by capillary sequencing 171 SNVs were also identified by the SOLiD sequencing, whereas two of the undetected SNVs were missed because less than 75% of the reads carried the non-reference allele. 33 of the tested SNVs were in the coding sequence of a gene and all these variants was identified in the SOLiD SNVs. Four of the undetected SNVs are in the hyper-polymorphic MHC region on chromosome 20. Further, no false positives were identified in these sequences.

### Variation analysis for the four genomes

#### SNVs

Each genome has about 3 million SNVs and 0.3-0.36 million indels compared to the BN reference. Approximately 70% of all SNVs are outside ref-seq annotated gene regions. About 1% of the SNVs are located in coding regions, and 21% of the SNVs in coding regions are non-synonymous (Table 1).

**Table 1:** **SNVs and short indels (< 10 bp) compared to the BN reference**

Strain	SNVs	SNVs in gene regions (± kb)	SNVs in cds	NS coding SNVs	Short indels	Indels in cds
DA/O	3.02 M	0.87 M	30440	6639	0.34 M	1206
DA/K	2.94 M	0.86 M	31130	6706	0.30 M	1243
E3	3.09 M	0.91 M	31426	6432	0.36 M	1294
PVG	2.99 M	0.86 M	31318	6432	0.30 M	1241

Cds = Coding sequence.

**Figure 3:**
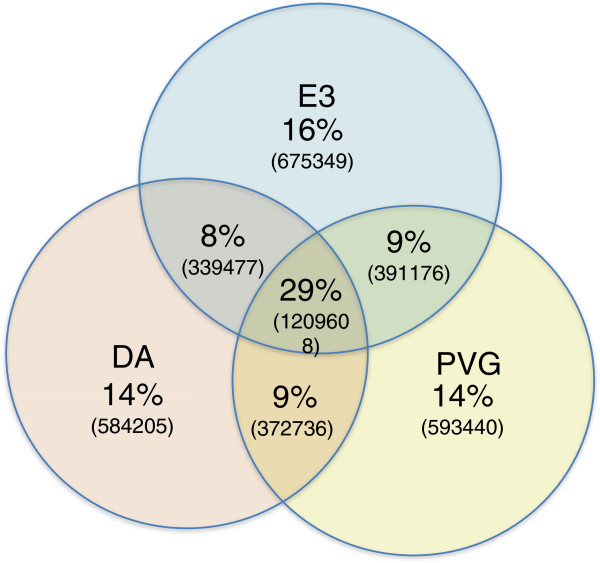
**Sequence similarities/differences between DA, E3 and PVG.** Similarity is given as the percentage of SNVs shared between combinations of strains out of all SNVs, after excluding 1.01 M positions where at least one strain is heterozygous or has a coverage below 3. The number of SNVs kept for the analysis was 4166021. Total numbers of SNVs are in parenthesis.

Pair-wise comparison identified that more than 98% of the high-quality (≥ 8X coverage) SNVs in DA/O are shared with the closely related DA/K. Analyzing the SNP distribution of the strains showed that about 14% of all SNVs identified are unique to DA/O and DA/K, compared to BN, E3 and PVG (Figure 3). Further, 29% of the identified SNVs are shared by all four genomes and differ only from the BN reference sequence. A more detailed distribution of the SNVs can be found in Table 2.

**Table 2:** **A summary of identified high-quality SNVs (coverage > 8) between, DA/K and DA/O, DA/K and PVG and DA/O and E3 and the distribution of SNVs across genomic features**

	DA/O≠DA/K	DA/O≠E3	DA/K≠PVG	DA/O+/K≠E3=PVG	BN≠DA/O+/K+E3+PVG
Stop gained	1	48	37	22	10
Stop lost	0	3	2	1	2
Non-synonymous	108	3688	3374	1628	1437
Synonymous	224	7398	7546	3193	2770
Essential splice site	1	41	36	23	20
splice site	21	1013	973	451	401
5’prime UTR	21	644	692	270	231
3’ prime UTR	113	4761	4481	2029	1860
Intronic	13949	436591	347893	180040	171296
Uppstream	1322	49342	40423	19954	18148
Downstream	1215	41414	34053	16472	14919

#### Stop codons

Each strain has between 70–79 SNVs that results in a predicted premature stop codon (Additional file 2). This affects a total of 131 genes, where about 60% are uncharacterized genes or olfactory and vomeronasal receptors. Further, in 32 of these genes the stop codon was predicted in an alternatively transcribed gene, and not affecting all annotated transcripts from the gene. 33 of the stop introducing SNVs were found in all 4 strains, whereas 56 are only present in one strain, with 23 unique for DA, 14 for PVG and 19 for E3. Furthermore, there is one stop gaining unique to the DA/K strain in the *Grb14* gene.

#### Structural variants

Next, we searched for structural variants between the strains. We detected 45 duplications in the range of 0.4-149 kbp, and 96 deletions between 1.8-134 kbp between DA/O and E3 (Additional file 3). Deletions were predicted to affect 96 genes in DA/O and 47 genes in E3. Fewer genes (42) were affected by deletions in DA/K compared to PVG and 36 genes in PVG compared to DA. The large number of gene deletions in DA compared to E3 is to a large extent due to deletions on chromosome 7 and 15, most of which are also deleted in PVG.

#### Correlating SNVs in different genomic features with differential expression and alternative splicing

Aiming to dissect the influence from SNVs occurring in different coding or transcript regulatory sequences such as splice-sites or UTRs, on differential expression and splicing, we compared the occurrence of SNVs within a certain gene with the expression of that gene. Intragenic SNVs were compared to a set of differentially expressed or alternatively spliced genes from a study by Gillett et al. [38]. In this study differential expression and alternative splicing was analyzed in RNA from lymph node cells from DA/K and PVG rats 7 days after induction of EAE. They detected 13 genes with convincing evidence of differential splicing between DA and PVG, of which three (Prex1, Itpr2 and Nab1) have predicted splice site variants that are unique to PVG. Further, 11 had coding SNVs. Naturally; it needs to be further investigated if these are the variants that lead to the altered splicing.

**Figure 4:**
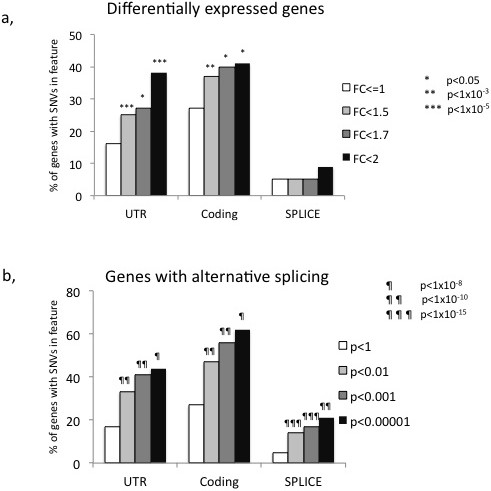
**The association of SNVs in different gene regions and differential expression or splicing.** Association of SNVs in different gene regions and differential expression or splicing, as reported in Gillett et al. [38]. The fractions of genes that have SNVs in i) coding region, ii) UTR or iii) splice sites were measured. **a,** Genes predicted to be differentially expressed between DA/K and PVG compared to genes with no differential expression in this condition. Three different levels of fold change were used to categorize differentially expressed genes, resulting in 88 genes with fold change (FC) > 2, 214 genes with FC > 1.7, 520 genes with FC > 1.5 and 14140 genes with no differential expression. **b,** Alternatively spliced genes compared with genes with no evidence of alternative splicing in this condition. Three significance levels were used to categorize significant alternative splicing in genes, resulting in 123 genes in the highest significance category p < 0.00001, 278 (p < 0.01), 613 (p < 0.1) and 13208 genes that were not considered alternatively spliced.

To assess the correlation between differential expression or splicing and SNVs on a larger scale, we looked for SNVs in coding or UTR regions as well as in splice sites in all genes with differential expression or splicing between DA/K and PVG in the Gillett et al. study and compared this with how often such SNVs occur in genes that were not differentially expressed or spliced. There was a significant enrichment of genes with SNVs in UTRs and coding sequences among the genes that displayed differential expression compared to genes that did not (Figure 4a). Due to the multiple probe design of the Affymetrix arrays, the coding SNVs should not influence the actual hybridization to the array and thus the difference should be reflecting the biology [39]. Similarly, there was an enrichment of genes with SNVs in splice sites, UTRs and coding regions in alternatively spliced genes compared to the genes where no alternative splicing was detected (Figure 4b). This suggests that coding SNVs as well as SNVs in UTRs and in splice sites are more frequent in genes that display differential expression or alternative splicing. However, there are still many genes without the variants that are differentially regulated. Hence, the absence of such variants cannot be used to exclude a gene as a candidate. In addition, there are additional types of genomic variations, such as larger structural variants like partial and whole gene duplications, which also need to be analyzed, since they can have a major impact on gene expression and splicing [40].

### Pairwise comparison of disease susceptible and resistant genomes

#### Comparing two DA sub-strains

**Figure 5:**
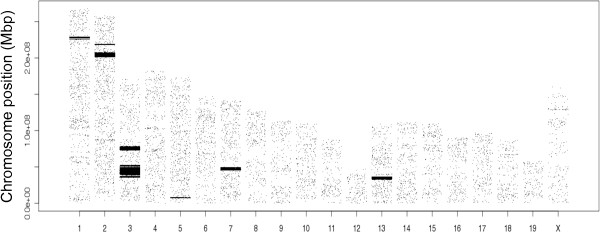
**DA/O and DA/K segregating SNV regions.** SNVs between DA/O and DA/K plotted on the chromosomes. The dark bands are due to high SNV density in 8 regions that distinguish DA/O and DA/K. Only positions supported by at least 8 reads in both strains were included in the picture.

There are more than 1 million of the identified SNVs between DA and E3 and between DA and PVG that could contribute to the phenotypic difference. However, even between the very similar DA/O and DA/K there is a difference in sensitivity to arthritis induction [13]. Looking closer at the polymorphic regions between DA/O and DA/K we could show that the strains differ primarily in regions on chromosomes 1, 2, 3, 7 and 13, which is in complete agreement with the results by Rintisch et al. However, we were able to better define the intervals, and two of the regions could be divided in two distinct regions each and we also found a new region on chromosome 5 that differs between these sub-strains (Figure 5 and Table 3). In total, these eight regions include approximately 38 Mbp and contain 302 genes of which 78 have nsSNVs or stop codon variants. Congenic data showed no effect on disease sensitivity from the region on chromosome 3 [13], hence the variants(s) causing the different arthritis susceptibility are more likely to be located on the other chromosomes, harboring in total 122 genes (Table 3).

**Table 3:** **DA/O and DA/K differentiating regions**

Chr.	Start	End	DA/O similar to:	DA/K similar to:	SNVs in region	Genes in region	Genes with NS SNVs	Polymorphic genes
1	225.91	229.22	ACI	F344, BUF	3871	21	2	Kank1, ENSRNOG00000023843.
2	201.5	207.21	LE	ACI	9084	74	14	Dennd2d, Prok1, Slc16a4, Gstm7, Clcc1, Prmt6.
	218.22	219.01	ACI, E3	F344, BN	1482	6	3	Arhgap29, Abca4, Dnttip2.
3	34.7	52.32	ACI	* (F344)	24496	80	20	Slc39a11, Galnt5, Upp2, L:y75, Dpp4, Grb14 (Stop).
	73.21	78.08	ACI, F344, BUF	* (WKY)	6287	100	33	C1qtnf4, Mybpc3, Ol726 (Stop), Acp2, Pacsin, Dgkz.
					Olfactory:	56	26	
					Other:	44	7	
5	7.1	8.06	*	*	1123	1	1	Depdc2 (Prex2)
7	45.76	48.5	SHR, F344, WKY	ACI	4352	13	3	Ptprq, ENSRNOG00000030316 (Otogl).
13	33.3	35.6	ACI, SHR	PVG, E3, BUF	5250	7	2	ENSRNOG00000030901, Dpp10.

(* unclear).

To infer if the regions of differences are contaminations from earlier crosses or hyper polymorphic regions, we compared the SNVs in the regions with the STAR genotypes of a number of other strains [37]. SNV comparisons show that DA/K or DA/O displays an alternating pattern of identical sequence to ACI in each of the identified intervals (Table 3). The patchy pattern of the diverging regions between the two DA strains is most likely the effect of the strains being separated and inbred separately before the DA strain was completely homozygous for all chromosomes.

The origin of the DA strain is often reported to be unknown but it has been documented to be derived from a stock from a Dr. T.T. Odell, Jr. at Oak Ridge National Laboratory, Tennessee. In 1957 Dr. Odell describes an agglutination test with an experimental set up using animals from a cross between two inbred lines that fits very well with what we now know about the DA strain [41]. The first strain for the initial intercross is an Irish coated strain that had been backcrossed for 47 generations that express the D antigen on their erythrocytes. This strain was most likely ACI (see Table 3). The second inbred strain in the inter-cross expressed the C antigen instead and had only been backcrossed for nine generations. Some suggestions of the C antigen strain are early generations of the E3, BUF or LEW strains, which carry this allele. The F1 progeny was then backcrossed to either the D or the C strain. The D/D backcross population is most likely the founder of the D-antigen expressing Agouti coat color (i.e. DA) strain. Inbreeding was at inter-cross generation 19 in about 1965. However a subset of the DA colony was sent from Wistar to Oxford already in 1963, where it was further inbred. Thus, the two DA sub-strains were separated at generation <19 before the chromosomes had become homozygous which explains the dissimilar regions. It is also possible that more DA sub-strains exist in laboratories throughout the world.

### Functional comparison – genomic variance within inflammation associated QTLs

Dissecting the genomic influence on inflammation requests a lot of phenotypic data. Both linkage studies and congenic mapping have identified ~20 genomic regions in the DA rat associated to inflammatory disease (Figure 2). The variation analyses of smaller QTLs, approximately 1 Mb in size, are described in more detail below and a schematic overview of all inflammation QTLs identified in comparisons of the three genomes irrespective of interval size are described in Table 4.

**Table 4:** **A summary of all coding SNVs in EAE and arthritis regulating QTLs**

Chr.	Phenotype/QTL	Start	End	QTL length (Mb)	Ensmbl genes in QTL	All genes with cSNVs	NS SNVs	STOP c. SNVs	Splice site SNVs	Indels	New candidate genes	Evidence	Strains	Reference
1	*PIA8*	11	19	8	43	3	2	0	0	1	NS SNVs in Ect2l, and ATG4B, indel in ALDH8A1	F2	DA.PVG,	[42]
1	*Eae29*	15	17	3	18	2	1	0	0	1	conserved SNVs in IL22RA2, short indel in ALDH8A1, NS SNV in ATG4B	G10, congene	DA/PVG	[29, 31]
1	*Eae30*	129	130	1	7	1	1	0	0	0	RGMA, NS SNV in Top1	G10, congene	DA/PVG	[29, 33]
1	*CIAA6/EAE6/7*	156	247	91	1343	225	136	2	93	12	stopcodon in Ltbp3 and in Ms4a5	F2 BC	DA/E3	[16]
1	*Eae6*	176	218	42	764	147	84	1	62	6	stopcodon in Ms4a5	F2	DA/E3	[18]
1	*Eae31*	185	185	1	6	3	2	0	1	0	IL21R 1 cons SNV, splice site in IL4R, NS SNVs in Gtf3c and LOC361646	G10, congene	DA/PVG	[15, 29, 33]
1	*Eae7*	240	247	7	64	25	16	0	12	1	indel in Myof, splice site SNV in Znf518a	F2	DA/E3	[18]
4	*PIA2/AIA2*	32	42	10	28	7	6	0	3	2	indel in Dpp6, and frameshift SNV in Tmem106b	F2 BC	DA/BN, E3	[8, 16]
4	*Eae24*	60	75	15	146	28	21	1	9	0	stop in an orf in a intron of Dgki	G10, congene	DA/PVG	[21, 43, 44]
4	*Eae25*	75	83	8	88	22	19	0	6	1	indel in Znf282 (HUB1)	G10, congene	DA/PVG	[21, 43, 44]
4	*PIA5/CIA3*	78	104	26	248	56	45	2	9	2	2 Stop in Igk genes	F2 BC congene	DA/BN,E3	[15, 16]
4	*Eae26*	83	104	21	206	28	24	0	5	2	indel in Herc6, and RPS7	G10, congene	DA/PVG	[21, 43, 44]
4	*Eae27*	104	114	10	61	7	6	0	1	0	NS SNVs in both CD8a and Cd8b	G10,congene	DA/PVG	[21, 43, 44]
4	*Eae20*	156	161	5	88	24	18	2	8	1	indel in Rad52, stop in Clec4b2, stop in Q6QI20	G10, congene	DA/PVG	[24, 44]
4	*PIA7/OIA2/CIA13*	159	160	1	23	8	6	2	2	0	stop in Clec4b2	F2 congene	DA/E3,PVG	[45, 46]
4	*Eae21*	161	162	1	23	4	2	0	3	0	splice SNV in Pianp, Zfp384, NS SNV Vwf	G10,congene	DA/PVG	[24, 44]
4	*Eae22*	162	172	10	137	34	27	1	14	1	Essential splice site SNV in Klrc2, stop Clec2	G10, BC	DA/PVG	[24, 44]
5	*CIAA5*	138	172	34	554	113	92	1	27	4	Stop in orf, indel in Nmnat1, Myom3, SZT2	F2 BC	DA/E3	[15, 16, 18]
6	*PIA3/eae9*	117	144	31	249	70	55	1	16	6	stop in F1M0Z7_RAT	F2 congene	DA/E3	[15, 16, 18]
7	*PIA13*	62	78	16	116	15	12	0	2	1	indel in Ptdss1	F2	DA/E3	[15, 16, 18]
8	*PIA14*	79	87	8	53	11	8	0	3	0	splice site in Lrrc1, Ick, Eef1a1	F2	DA/E3	[15, 16, 18]
10	*Vra4/Eae*	0	11	11	94	13	11	0	2	0	no coding SNVs in Ciita	congene	DA/PVG	[47, 48]
10	*Eae18a*	55	67	12	303	67	47	1	20	2	stop in krt18-b, 2 NSC and splice site SNV in Nos2	G7,G10,congene	DA/PVG	[20, 22, 25]
10	*Eae18b*	67	72	5	68	22	12	0	10	2	CCL11 conserved SNVs	G7,G10,cong.	DA/PVG	[20, 25, 30, 49]
10	*CIA5A OIA3 PIA10 EAN5*	95	110	15	214	36	29	0	8	3	Cd300 hyper polymorphic regions, indel in Fdxr, Acox1, Tnrc6c	G10 F2 congene	DA/PVG	[14, 50]
11	*Pia27, RF*	84	86	2	53	6	4	0	2	1	Essential splice site in Lac2	F2 congene	DA/E3	[51]
12	*Eae5*	20	24	4	70	16	11	0	5	0	NCF1, CLN4, splice site SNV i Gtfi1	G7, congene	DA/PVG	[28, 49]
12	*PIA4/NCF1*			*			1				NCF1 NS SNV aa153	congene	DA/E3	[16, 52]
14	*PIA6/EAE10/CIAA7*	32	103	71	408	39	25	0	12	3	NS SNV Tlr10, Igfbp3, indel Aebp1, Apex2	F2	DA/E3	[53, 54]
15	*Eae19*	83	97	14	33	7	7	0	1	0	Lmo7, Tbc1d4, Mycbp2, Uchl3, Slitrk6	G7,congene	DA/PVG	[20, 26]
17	*Eae23a*	36	57	21	180	13	12	0	1	0	NS SNVs in Ripk1, Serpinb1a, Gpx6, splice-SNV in Agtr1a	G10, cong.	DA/PVG	[29, 32, 55]
17	*Eae23b*	57	66	9	49	11	8	1	3	1	stop in Prelid1	G10, cong.	DA/PVG	[29, 32, 55]
20	*Pia1*	3	5	2	114	50	40	1	16	0	NS SNVs in Ncr3, Lst1, RT1-Ba, Tap2, Psmb8, Tap1, Psmb9, Stop in RT1-CE4	F2	DA/E3, PVG DA/LEW,BN	[8, 15]
20	*Eae1*	5	7	2	55	14	8	0	4	3	NS SNVs in Tapbp, RT1-A2, RT1-A2 frameshift mutation in Grm3	F2	DA/E3, PVG DA/LEW,BN	[18]

#### Chromosome 1

Chromosome 1 harbors two regions of QTLs for both EAE and PIA, [16, 18, 29, 31, 33, 42], The fine-mapping of the two QTLs down to a shorter genomic interval has been done in EAE.

**Figure 6:**
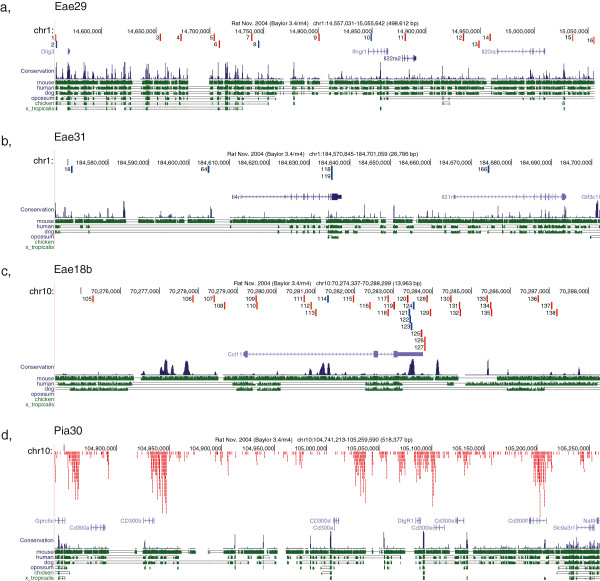
**Snapshots from the UCSC genomic web-browser describing the genomic locations of the identified SNVs within 4 QTLs or specific genes in QTLs (a-d).** The SNVs are depicted as red or blue bars in the top of the illustration. The number next to the bar corresponds to the SNPs individual number in the series of all SNPs in the QTL. Blue bars indicate SNVs with conservation score higher than 0.1, in red are SNVs less that 0.1. The mid segment of the snapshot contains the genes in the interval and below is the degree of inter-species conservation illustrated as blue and green bars.

One QTL region *Eea29/Pia8* is positioned close to the centromere and this region regulates both EAE and PIA. Beyeen et al. fine-mapped this QTL in EAE to less that 3 Mb using an AIL and they also validated the regulatory effect using a 25 Mb congenic strain [31]. The refined *Eae29/Pia8* contains 18 genes. Beyeen et al. could show differential gene expression in the genes *IL22RA2* and *IFNGR1* and association to *IL22RA2* could be shown in a cohort of MS patients. Therefore these two genes are strong candidates as regulators of inflammatory disease and thus it is important to study all variants in the region for regulatory effects and if they coincide with conserved regions. Comparing SNVs between the strains DA and PVG in the shorter *Eae29* region displayed very little variation between DA and PVG. Surprisingly, only 16 SNVs were identified between 14.5-15.1 Mb. *IL22ra2* had one SNV in the second intron and *Ifngr1* had one SNV in the first intron. To analyze if the SNV is in a conserved segment and thus may be coinciding with a regulatory element, the conservations score for the SNVs was assessed. The SNV is considered to be conserved if the score is higher than 0.1, (http://hgdownload.soe.ucsc.edu/goldenPath/rn4/phastCons9way/). Three of the SNVs in *Eae29* coincide with highly conserved regions (Figure 6a). Further comparison of these SNVs to data from eight inbred rat strains [56] identified two SNVs in conserved regions to be unique for the DA stain and the SNV in *Il22ra2* is unique for DA and one of its ancestral genomes ACI (Additional file 4).

The second QTL on chromosome 1 has been fine mapped in both EAE and PIA from an interval between 176–218 Mb and was resolved into two distinct QTLs *Eae30* and *Eae31. Eae31* is positioned to a region of less than 1 Mb around 185 Mb and Eae30 to 1 Mb close to 128 Mb [33]. *Eae31* was fine-mapped in both EAE and PIA to five candidate genes and the region showed evidence of association to human inflammatory disease to a SNV in the *IL21R*. No coding polymorphisms could be identified in the *IL21R* gene in the rat, but the *IL4RA* gene had a splice site SNV. Four SNVs are unique to DA (Additional file 4). In addition, two other genes in the region, *Gtf3c* and *LOC361646,* displayed numerous nsSNVs. Further, looking at SNVs in conserved regions identified 5 potentially regulating SNVs (Figure 6b). The *IL4RA* gene also contains two sSNVs, whereas the *IL21R* gene contains one potentially regulating SNV in an intron (conservation score 0.45).

In the Nohra et al. study [33], an additional 1.1 Mb EAE specific QTL *Eae30* close to the *RGMA* gene was identified in a G10 AIL. Interestingly, there was suggestive association to the human *RGMA* gene also in an MS cohort. There are 48 polymorphisms between DA and PVG in *RGMA*. Ten of the variants are of potential interests; three SNVs in conserved regions and one synonymous SNV in the last exon. Four SNVs in the region are unique for DA/ACI (Additional file 4).

#### Chromosome 4

Rat chromosome 4 is one of the most QTL dense regions in the DA genome. Three regions have been linked to EAE and arthritis. The QTL *Pia5/Eae24-27* have also been linked to experimental diabetes [57] and uveitis [58]. There is also evidence that the chromosome has regions of epistatic genetic interactions [43].

The original *Pia5/Eae24-27* locus is more than 20 Mb and covers 250 to 500 genes depending on the study and disease model [16, 43]. Among the genes in this region are *TCRBV, Ig Kappa* genes and *IL23R*, with potential profound importance in immune regulation. In 2010 Marta et al. used a combination of congenic lines as well as an advanced intercross line to dissect disease regulation in this region. They showed that the effect on disease regulation was larger than the effect from any of the smaller congenic strains, however one small (1.25 Mb) congenic strain (R23) mediates a major part of the regulatory effect from this region. No coding variations could be identified between DA and PVG in this region, which indicates that the disease regulating variation is regulatory. Indeed, this region is very conserved between species and of the 1810 SNVs in the interval 188 coincided with conserved genomic intervals. Not surprisingly, many of the genes in the region have important function in embryonic development.

Next, the arthritis QTL *Oia2/Pia7* at 159.46-160.0 Mb is known to be a strong regulator of adjuvant-induced arthritis such as OIA or PIA [42, 45, 46]. Further, this arthritis QTL coincides with the EAE QTLs *EAae20-21*[24]. The region harbors the APLEC complex, which encodes a set of C-type lectin receptor genes expressed on antigen presenting cells. Most of the APLEC genes have been shown to be differentially expressed after stimulation with a number of immune-triggering stimulants [45, 59]. There are a number of coding polymorphisms in the APLEC complex encoded genes. One generates a premature stop-codon in *Clec4b2* in the DA genome and is certainly a plausible candidate to mediate the disease phenotype. However, four other APLEC encoded genes have non-synonymous SNVs between DA and E3/PVG. Furthermore, a total of 832 SNVs could be identified in the 550 kb consensus region and 33 polymorphisms lie within conserved regions in or close to genes. Neither of the SNVs in the APLEC complex are unique to DA but are shared between at least five other inbred genomes (Additional file 4) [56]. One region at 159.955 kb has the maximum conservation score = 1, according to the phastCons9way, and could be a regional enhancer for the APLEC genes in the region. This region harbors two SNVs. This region needs to be analyzed further to conclusively determine weather this is an enhancer or not.

#### Chromosome 10

Chromosome 10 harbors at least four susceptibility genes for experimental inflammatory disease. Proximal to the centromere is the *Vra1*/*Ciita* gene locus [47, 48], which is part of a haplotype identified to regulate levels of MHC class II expression, and which was also reported to be associated with chronic inflammatory disease [48]. The variant underlying the effect has however not been identified. *Ciita* contains no nsSNVs between DA and PVG. However, there are three synonymous coding SNVs that may influence the expression levels of which two are unique for E3/PVG (Additional file 4). In addition, there are a number of polymorphisms in conserved regions with potentially regulatory effect on the gene. The 19 kb upstream promoter of *Ciita* harbors two very conserved regions with 10 SNVs of which one is unique to E3/PVG.

The mid-part of chromosome 10 has been associated with both EAE and arthritis *Eae18/Cia5/Oia3*[25, 50, 60]. *Eae18* QTL was fine-mapped using two AILs, G7 and G10, which divided the QTL in two QTLs, *Eae18a* and *Eae18b*[25]. *EAE18a* was fine-mapped to 1.1 Mb harboring 13 genes at 58.2-59.3 Mb [22]. Two nsSNVs in *Fam64a* and ENSRNOG00000037371, as well as two SNVs in splice-sites of RGD1304728 and *Smtln2* were identified in the *Eae18a* locus, in addition to several potentially regulating SNVs.

*Eae18b* was fine-mapped to a 0.88 Mb region between 70.2-71.0 Mb in a G10 AIL [49]. Two genes in this region *Ccl2* and *Ccl11* showed differential expression. Interestingly, differential expression of *Ccl2* has also been observed between MS patients and controls. However in a functional characterization study of the *Eae18b* congenic strain, *Ccl11* was conclusively identified to be regulating many different disease pathways in this strain [30]. *Ccl11* has six SNVs in the 3′UTR of which four are in conserved sequences; the gene also has one conserved intronic SNV and one synonymous SNV (Figure 6c).

In experimental arthritis a QTL has been identified in both DA/PVG and DA/F344 crosses on chromosome 10 [14, 50, 60]. This arthritis specific QTL was fine-mapped in pristine-induced arthritis using an AIL G10 [14]. Also for this QTL the locus was separated in to two regions. Interestingly the second 1.5 Mb region coincided with a region with Cd300 like genes, which contain distinct regions of very high variation between DA and the other strains (Figure 6d). The aggregation of SNVs in the vicinity of *Cd300* genes may be an indication of structural variations in the region and needs to be further studied, however these genes could have a significant influence on the regulation mediated by this region. Interestingly this locus was not mapped in any of the DAxE3 F2 crosses, which suggests that the regulating polymorphism(s) could be unique to the PVG strain. In the other region Pia30, 114 SNVs were identified that segregated E3/DA and PVG alleles, of which most are intronic or intergenic SNVs in or near the *Rpl38* and *Ttyh2 genes.*

#### Chromosome 12

The first arthritis regulating QTL to be cloned and positioned to one polymorphism is the Pia4, which was identified to be regulating chronic inflammation by a non-synonymous SNV in the *Ncf1* gene, altering the amino acid at position 153 [52]. It was originally mapped using both PIA (*Pia4*) [8] and EAE models (*Eae5/Eae11*) [18, 23]. The gene was identified using a congenic strain with E3 alleles between 23.476-23.730 Mb and harbors three genes. Here, a total of 377 SNVs were detected between DA and PVG/E3 in the *Pia4* region. Five of them are coding variants, of which one is non-synonymous and one is annotated to be in a splice site of *Gtf2i*. Moreover, 7 SNVs are located in UTRs of which 5 are in the 3′UTR of the *Ncf1* gene, but they have low conservation scores. Five SNVs are in potentially conserved regions, but only one of them, the nsSNV, is in *Ncf1*. Hence, the genome sequences support the previous findings that the inflammation regulatory effect from this congenic is mediated by the *Ncf1* nsSNV [61].

## Conclusions

DA rats are particularly susceptible to the induction of a number of chronic inflammatory diseases. There is a modest difference in arthritis susceptibility between the two strains where DA/O is slightly more susceptible. Here we identified eight polymorphic regions between two substrains of DA; DA/K and DA/O together covering 38 Mb of genomic sequence. Of the variable regions, 23 Mb are located on chromosome 3 and by using a DA/O.chr3DA/K congenic strain these regions were excluded as important in arthritis regulation [13], and thus the regulatory effect should come from the remaining 15 Mb variable regions between DA/O and DA/K.

According to the rat genome database more that 50 inflammatory disease related QTLs have been identified in the DA rat. Here we have used the next-generation sequencing of the DA genome and inflammatory disease resistant E3 and PVG to investigate 10 fine-mapped QTLs and closely catalogue the complete set of variations in these regions. This study has identified a number of interesting non-synonymous or regulatory variants within these QTLs.

For the already positionally cloned gene, *Ncf1,* in the *Pia4* QTL on chromosome 12, no additional regulatory SNVs were identified in the proximal region of *Ncf1* that are likely to contribute to the regulation of inflammation in the region. Hence, this further enhances the importance of the previously detected nsSNV (coding for aa153 of the protein) in the regulation of experimental inflammatory disease.

Two of the inflammatory-disease regulating regions analyzed here; the *Vra1/Ciita* and *Eae25*/R23 loci are completely devoid of coding SNVs, but do contain several SNVs in conserved regions, indicating regions of regulatory function.

Four of the QTLs (*Eae29, Eae30, Eae31* and *Eae18b*) have strong candidate genes that have been identified to be differentially expressed between DA and PVG as well as associated to chronic inflammatory disease in human cohorts. These gene-regions were analyzed for regulatory polymorphisms*. Eae29* has three very good regulatory SNV candidates that should be assessed for disease regulation in the vicinity of *IL22ra2.* The expression of *IL22ra2* has been demonstrated to differ between the strains implicating difference in regulatory regions [31]. *IL22RA2* is now one of the well-established MS risk genes [62]. *Eae30* has one non-synonymous SNV in a topoisomerase like gene, however this gene may not be the disease-regulating gene in the interval. *RGMA*, which is the top candidate gene in the region, contains 4 conserved regions with possible regulatory SNVs. The top candidate gene for the *Eae31* locus is *Il21r*. This gene has only one identified SNV with potential regulatory function in the first intron of the gene. *Eae31* also contains a splice site SNV in the I*l4ra* gene plus two potential regulating SNVs that also should be tested for EAE regulation. The QTL *Eae18b* harbors the *Ccl11* gene that have shown strong immune-regulatory effects in inflammatory disease [30]. *Ccl11* contains five highly conserved SNVs and particularly four SNVs in the 3′UTR are strong candidates to be regulators of differential expression.

The APLEC locus on chromosome 4 harbors five nsSNVs in four genes. This region contains very few conserved regions, however a new impending regulatory region 50 kb upstream of the last gene (*Mincle*) in the complex was identified that contains two SNVs in the middle of the conserved region. This region has to be analyzed further to fully comprehend if and how it could regulate all genes in the region.

A hyper-variable region in *Pia30* on chromosome 10 was also identified. This region contains four SNV dense regions of about 10 kb in size each. These variable regions occur in the extended 3′UTR of *Cd300* genes, *Cd300b* and *Cd300f* in particular. This enrichment of SNVs in a short interval may perhaps be explained by sequence duplications/divergence.

By combining information for the most interesting SNVs in the herein analyzed QTLs with SNV information from an eight genome sequencing study of inbred rats [56], we identified a number of SNVs unique for inflammation susceptible DA and the inflammatory disease resistant PVG and E3 strain. Three QTLs had DA or DA/ACI unique SNVs and one had E3/PVG unique SNVs. Out of the eight QTLs that were used in this comparison only four had unique SNVs. This is somewhat expected since one of the few cloned chronic inflammatory disease regulating SNV, in the Ncf1 gene, also is present in the inbred strains BUF, F344 and WKY. The strain BUF is susceptible to both arthritis and EAE whereas both F344 and WKY are resistant to experimental arthritis. The disease regulation mediated by this SNV is however very strong and has been confirmed to be disease regulating also in mice. This demonstrates that additional protective genes in the genome may mask even very strong disease enhancing SNV effects.

In a genotype to phenotype perspective, the combined effect from the QTLs has profound functional implications. In short, both the adaptive and innate immunity is affected; The Ncf1 gene is involved in the first defense against infections and triggering the immune system. Both the CD300 and the APLEC encoded genes are important pattern recognition receptors involved in antigen uptake and Ciita is important in regulating antigen presentation. The chemokine Ccl11 and the cytokine receptors Il21r and Il22ra2 are important in modulating the immune responses leading to inflammation. Il21r and Il22ra2 are particularly important regulators of activated T cells. In conclusion the 10 QTLs represent regions of important disease regulation and the combined effect explains a substantial part of the pro-inflammatory phenotype that is characteristic of the DA strain.

The full genomic sequence of DA/O, DA/K, E3 and PVG constitutes an invaluable resource in the understanding of how polymorphisms in the DA genome contribute to disease susceptibility and this genetic blueprint of an inflammation permissive rat strain will also be important in the understanding of the human etiologies for chronic inflammatory disease.

## Methods

### Next-generation sequencing

Liver samples from 1 female rat per strain was used for all runs. The sequences were generated in 3 separate runs. The first run (august 2009) was achieved using a SOLiD™3 sequencer with 1 full slide per sample. The Mate-pair library protocol was used to generate 2 × 50 bp reads, with 2–3 kb insert size. A Hydro-shear was used for fragmentation. The libraries were produced after 11 cycles of amplification. The second run (September 2010) was performed using a SOLiD™4 and generating 2 Mate-pair libraries per genome, one with 1 kb insert size and one with 4–4.5 kb insert sizes. ¼ of a slide was used for each library and the libraries were amplified 10–15 times. The DNA samples were fragmented using a Covaris S2. The last sequencing run using the Fragment library protocol was done on a SOLiD™5500 (June 2011). 3 lanes of 75 X 35 bp reads were run per sample. Fragmentation to 150–200 bp was achieved by a Covaris S2 with 6 cycles of amplification. All libraries in the study were quality controlled on an Agilent Bioanalyser High sensitivity Chip + qPCR.

### Mapping

The reads were aligned to the BN reference assembly (Rnor3.4) with the BWA (v0.5.9) aligner (−c –l 25 –k 2 –n 10). Mapped reads from all libraries were merged before SNV calling. The yield of sequence mapping to the genome was 48.9 Gb for DA/O, 50.8 Gb for DA/K, 59.9 Gb for E3/han and 51.8 Gb for PVG/1AV1.Kini (Additional file 1).

### SNV/Indel calling

SNV and short indel calling was done with a strategy developed by Guryev [36] based on Samtools pileup [63]. Duplicate reads (starting at the same position) were discarded. Next, each SNV should be supported by at least three calls with a quality > 10. In addition, we used only SNVs where at least 75% of the reads support the non-reference allele (40% for indels). Finally, SNVs that differed between the reference and the resequencing of the original reference rat (Eve), were removed since these are likely to be errors in the reference. This resulted in a total of 5.2 M SNVs and 0.66 M short indels (< 10 bp), of which about 57% were deletions and 43% insertions. A “high-quality SNV set” was compiled of SNVs with at least 8 X coverage. About 97% of the genome was covered by at least 3 calls and 85% by 8 or more.

The functional annotations of all SNVs were predicted with the Ensembl Variant Effect Predictor [64].

These strains have been inbred for many generations and should be homozygous at nearly all positions. Therefore we excluded approximately 0.9 M positions per strain where 25-75% of the reads supported the variant allele. Almost 10% of these variants are detected in regions with high coverage (>30X), compared to <1% for homozygous SNVs, and may be due to duplications, as suggested previously [40]. Further, we found that 7% of the heterozygous SNVs in DA/O (coverage 9-25X) were called as homozygous SNVs in DA/K, whereas 1% were called as reference. Hence, some heterozygous SNV calls are likely true SNVs which are missed due to low coverage and errors in sequencing/alignment.

To estimate the SNV calling sensitivity we compared our SNVs with a set of 20284 SNVs typed by the STAR consortium [37]. In this set, 11241, 11037 and 11253 SNVs differed between BN and DA, E3 and PVG, respectively, of which, we detected between 96.8-97.8% in all strains. We also sequenced a number of SNV dense regions with conventional Sanger sequencing. Out of the 178 SNVs detected by Sanger sequencing, 171 could be detected by the SOLiD sequencing, whereas two were predicted by less than 75% of the reads and thus were false negatives. No false positives were found with SOLiD in these regions.

The sensitivity of indel calling could not be estimated using STAR genotypes and the Sanger sequenced regions did not contain any indels, however it is likely that the sensitivity is substantially lower. Indeed, we found that only 66% of the indels called in DA/O were confirmed in DA/K.

### CNV calling

Copy number variation (CNVs) due either to deletions or duplications of regions. were predicted with DWAC-seq version 0.56 to identify CNVs. This program counts the reads in a window of dynamic size and compares these counts for two strains. We counted only uniquely mapped reads and used a ratio below 0.3 for deletions and above 1.6 for duplications. To better distinguish CNVs from repetitive regions, we only allowed regions with one strain having a mean coverage around the mean genome coverage (±0.5* mean coverage). Further, regions were only predicted as deletions if the number of bases lacking coverage differed by at least 10% between the strains.

### Ethics statement

All experiments in this study were approved and performed in accordance with the guidelines from the Swedish National Board for Laboratory Animals and the European Community Council Directive (86/609/EEC) under the ethical permit N332/06entitled ‘Genetic regulation, pathogenesis and therapy of EAE, an animal model for multiple sclerosis’, which was approved by the North Stockholm Animal Ethics Committee (Stockholm snorra djurförsöksetiska nämnd). Rats were tested according to a health-monitoring program at the National Veterinary Institute (Statens Veterinärmedicinska Anstalt, SVA) in Uppsala, Sweden.

### QTL analysis

QTLs smaller than 1 Mb in interval were selected for finer SNV analysis. SNVs were visualized using the add-custom track function in the UCSC web browser. The conservation scores (phastCons9way) where downloaded from the UCSC. http://hgdownload.soe.ucsc.edu/goldenPath/rn4/phastCons9way/. In order to retrieve information on unique SNVs in the smallest QTLs, conserved SNVs or nsSNVs in the QTLs were compared to eight inbred rat strains [56] in a 13 genome comparison.

### Accession numbers

The sequence data for all four genomes will be available from the European Bioinformatics Institute short read archive under the accession number: ERP0004908. All variants identified in this study will be available from the rat genome database (http://rgd.mcw.edu).

## Electronic supplementary material

Additional file 1: **Yield and coverage for sequence libraries.** Table describing the different libraries that were created, and the yield and genome coverage from sequencing. (XLSX 43 KB)

Additional file 2: **Stop-gained or stop-lost nsSNVs.** Table with nsSNVs that generates stop-gained or stop-lost. (XLSX 66 KB)

Additional file 3: **Insertions and deletions.** Table describing insertions and deletions identified in the sequencing study. (XLSX 46 KB)

Additional file 4: **A 16 rat strain SNV analysis within eight inflammation associated QTLs.** Table including SNV information for 9 rat strains [56] within eight of the smallest QTLs discussed in the Results section. The SNV that are included in the table are all identified nsSNVs, sSNVs splice-site variants and conserved SNVs. (XLSX 55 KB)

## Abbreviations

Chr: Chromosome
PIA: Pristane-induced arthritis
EAE: Experimental autoimmune encephalomyelitis
OIA: Oil-induced arthritis
DA/O: DA/OlaHsd DA/K, DA/hanKini
QTL: Quantitative trait locus
SNV: Single nucleotide variation
SY-SNV: Synonymous SNV
NS-SNV: Non-synonymous SNV
cSNV: SNV in coding region.

## References

[1] Atanur SS, Birol I, Guryev V, Hirst M, Hummel O, Morrissey C, Behmoaras J, Fernandez-Suarez XM, Johnson MD, McLaren WM, Patone G, Petretto E, Plessy C, Rockland KS, Rockland C, Saar K, Zhao Y, Carninci P, Flicek P, Kurtz T, Cuppen E, Pravenec M, Hubner N, Jones SJM, Birney E, Aitman TJ (2010). The genome sequence of the spontaneously hypertensive rat: analysis and functional significance. Genome Res.

[2] Seldin MF, Amos CI, Ward R, Gregersen PK (1999). The genetics revolution and the assault on rheumatoid arthritis. Arthritis Rheum.

[3] Hauser SL, Oksenberg JR (2006). The neurobiology of multiple sclerosis: genes, inflammation, and neurodegeneration. Neuron.

[4] Fernando MMA, Stevens CR, Walsh EC, De Jager PL, Goyette P, Plenge RM, Vyse TJ, Rioux JD (2008). Defining the role of the MHC in autoimmunity: a review and pooled analysis. PLoS Genet.

[5] Manolio TA, Collins FS, Cox NJ, Goldstein DB, Hindorff LA, Hunter DJ, McCarthy MI, Ramos EM, Cardon LR, Chakravarti A, Cho JH, Guttmacher AE, Kong A, Kruglyak L, Mardis E, Rotimi CN, Slatkin M, Valle D, Whittemore AS, Boehnke M, Clark AG, Eichler EE, Gibson G, Haines JL, Mackay TFC, McCarroll SA, Visscher PM (2009). Finding the missing heritability of complex diseases. Nature.

[6] Trentham DE, Townes AS, Kang AH (1977). Autoimmunity to type II collagen an experimental model of arthritis. J Exp Med.

[7] Holmdahl R, Goldschmidt TJ, Kleinau S, Kvick C, Jonsson R (1992). Arthritis induced in rats with adjuvant oil is a genetically restricted, alpha beta T-cell dependent autoimmune disease. Immunology.

[8] Vingsbo-Lundberg C, Nordquist N, Olofsson P, Sundvall M, Saxne T, Pettersson U, Holmdahl R (1998). Genetic control of arthritis onset, severity and chronicity in a model for rheumatoid arthritis in rats. Nat Genet.

[9] Lorentzen JC, Andersson M, Issazadeh S, Dahlman I, Luthman H, Weissert R, Olsson T (1997). Genetic analysis of inflammation, cytokine mRNA expression and disease course of relapsing experimental autoimmune encephalomyelitis in DA rats. J Neuroimmunol.

[10] Dahlman I, Wallström E, Jiao H, Luthman H, Olsson T, Weissert R (2001). Polygenic control of autoimmune peripheral nerve inflammation in rat. J Neuroimmunol.

[11] Sun B, Sun SH, Chan CC, Caspi RR (2000). Evaluation of in vivo cytokine expression in EAU-susceptible and resistant rats: a role for IL-10 in resistance?. Exp Eye Res.

[12] Rose NR (1975). Differing responses of inbred rat strains in experimental autoimmune thyrioditis. Cell Immunol.

[13] Rintisch C, Holmdahl R (2008). DA rats from two colonies differ genetically and in their arthritis susceptibility. Mamm Genome.

[14] Bäckdahl L, Guo JP, Jagodic M, Becanovic K, Ding B, Olsson T, Lorentzen JC (2009). Definition of arthritis candidate risk genes by combining rat linkage-mapping results with human case–control association data. Ann Rheum Dis.

[15] Olofsson P, Holmberg J, Pettersson U, Holmdahl R (2003). Identification and isolation of dominant susceptibility loci for pristane-induced arthritis. J Immunol.

[16] Olofsson P, Lu S, Holmberg J, Song T, Wernhoff P, Pettersson U, Holmdahl R (2003). A comparative genetic analysis between collagen-induced arthritis and pristane-induced arthritis. Arthritis Rheum.

[17] Holmberg J, Tuncel J, Yamada H, Lu S, Olofsson P, Holmdahl R (2006). Pristane, a non-antigenic adjuvant, induces MHC class II-restricted, arthritogenic T cells in the rat. J Immunol.

[18] Bergsteinsdottir K, Yang HT, Pettersson U, Holmdahl R (2000). Evidence for common autoimmune disease genes controlling onset, severity, and chronicity based on experimental models for multiple sclerosis and rheumatoid arthritis. J Immunol.

[19] Yang HT, Bergsteinsdottir K, Wernhoff P, Linington C, Pettersson U, Holmdahl R (2001). Genetic linkage analysis of the antibody responses to myelin basic protein and myelin oligodendrocyte glycoprotein in rats immunized with rat spinal cord homogenate. J Neuroimmunol.

[20] Dahlman I, Wallström E, Weissert R, Storch M, Kornek B, Jacobsson L, Linington C, Luthman H, Lassmann H, Olsson T (1999). Linkage analysis of myelin oligodendrocyte glycoprotein-induced experimental autoimmune encephalomyelitis in the rat identifies a locus controlling demyelination on chromosome 18. Hum Mol Genet.

[21] Dahlman I, Lorentzen JC, de Graaf KL, Stefferl A, Linington C, Luthman H, Olsson T (1998). Quantitative trait loci disposing for both experimental arthritis and encephalomyelitis in the DA rat; impact on severity of myelin oligodendrocyte glycoprotein-induced experimental autoimmune encephalomyelitis and antibody isotype pattern. Eur J Immunol.

[22] Ockinger J, Serrano-Fernández P, Möller S, Ibrahim SM, Olsson T, Jagodic M (2006). Definition of a 1.06-Mb region linked to neuroinflammation in humans, rats and mice. Genetics.

[23] Becanovic K, Jagodic M, Sheng JR, Dahlman I, Aboul-Enein F, Wallström E, Olofsson P, Holmdahl R, Lassmann H, Olsson T (2006). Advanced intercross line mapping of Eae5 reveals Ncf-1 and CLDN4 as candidate genes for experimental autoimmune encephalomyelitis. J Immunol.

[24] Jagodic M, Marta M, Becanovic K, Sheng JR, Nohra R, Olsson T, Lorentzen JC (2005). Resolution of a 16.8-Mb autoimmunity-regulating rat chromosome 4 region into multiple encephalomyelitis quantitative trait loci and evidence for epistasis. J Immunol.

[25] Jagodic M, Becanovic K, Sheng JR, Wu X, Bäckdahl L, Lorentzen JC, Wallström E, Olsson T (2004). An advanced intercross line resolves Eae18 into two narrow quantitative trait loci syntenic to multiple sclerosis candidate loci. J Immunol.

[26] Sheng JR, Jagodic M, Dahlman I, Becanovic K, Nohra R, Marta M, Iacobaeus E, Olsson T, Wallström E (2005). Eae19, a new locus on rat chromosome 15 regulating experimental autoimmune encephalomyelitis. Genetics.

[27] Lorentzen JC, Glaser A, Jacobsson L, Galli J, Fakhrai-rad H, Klareskog L, Luthman H (1998). Identification of rat susceptibility loci for adjuvant-oil-induced arthritis. Proc Natl Acad Sci U S A.

[28] Lorentzen JC, Klareskog L (1997). Comparative susceptibility of DA, LEW, and LEW.1AV1 rats to arthritis induced with different arthritogens: mineral oil, mycobacteria, muramyl dipeptide, avridine and rat collagen type II. Transplant Proc.

[29] Becanovic K, Wallström E, Kornek B, Glaser A, Broman KW, Dahlman I, Olofsson P, Holmdahl R, Luthman H, Lassmann H, Olsson T (2003). New loci regulating rat myelin oligodendrocyte glycoprotein-induced experimental autoimmune encephalomyelitis. J Immunol.

[30] Adzemovic MZM, Ockinger JJ, Zeitelhofer MM, Hochmeister SS, Beyeen ADA, Paulson AA, Gillett AA, Hedreul MMT, Covacu RR, Lassmann HH, Olsson TT, Jagodic MM (2011). Expression of Ccl11 associates with immune response modulation and protection against neuroinflammation in rats. PLoS ONE.

[31] Beyeen ADA, Adzemovic MZM, Ockinger JJ, Stridh PP, Becanovic KK, Laaksonen HH, Lassmann HH, Harris RAR, Hillert JJ, Alfredsson LL, Celius EGE, Harbo HFH, Kockum II, Jagodic MM, Olsson TT (2010). IL-22RA2 associates with multiple sclerosis and macrophage effector mechanisms in experimental neuroinflammation. J Immunol.

[32] Stridh PP, Hedreul MMT, Beyeen ADA, Adzemovic MZM, Laaksonen HH, Gillett AA, Ockinger JJ, Marta MM, Lassmann HH, Becanovic KK, Jagodic MM, Olsson TT (2009). Fine-mapping resolves Eae23 into two QTLs and implicates ZEB1 as a candidate gene regulating experimental neuroinflammation in rat. PLoS ONE.

[33] Nohra R, Beyeen AD, Guo JP, Khademi M, Sundqvist E, Hedreul MT, Sellebjerg F, Smestad C, Oturai AB, Harbo HF, Wallström E, Hillert J, Alfredsson L, Kockum I, Jagodic M, Lorentzen J, Olsson T (2010). RGMA and IL21R show association with experimental inflammation and multiple sclerosis. Genes Immun.

[34] Raymond SL, Dodds WJ (1975). Characterization of the fawn-hooded rat as a model for hemostatic studies. Thromb Diath Haemorrh.

[35] Li H, Durbin R (2009). Fast and accurate short read alignment with Burrows-Wheeler transform. Bioinformatics.

[36] Guryev V, Saar K, Adamovic T, Verheul M, van Heesch SAAC, Cook S, Pravenec M, Aitman T, Jacob H, Shull JD, Hubner N, Cuppen E (2008). Distribution and functional impact of DNA copy number variation in the rat. Nat Genet.

[37] Saar K, Beck A, Bihoreau M-T, Birney E, Brocklebank D, Chen Y, Cuppen E, Demonchy S, Dopazo J, Flicek P, Foglio M, Fujiyama A, Gut IG, Gauguier D, Guigo R, Guryev V, Heinig M, Hummel O, Jahn N, Klages S, Kren V, Kube M, Kuhl H, Kuramoto T, Kuroki Y, Lechner D, Lee Y-A, Lopez-Bigas N, Lathrop GM, Mashimo T, STAR Consortium (2008). SNP and haplotype mapping for genetic analysis in the rat. Nat Genet.

[38] Gillett A, Maratou K, Fewings C, Harris RA, Jagodic M, Aitman T, Olsson T (2009). Alternative splicing and transcriptome profiling of experimental autoimmune encephalomyelitis using genome-wide exon arrays. PLoS ONE.

[39] Huang G-J, Shifman S, Valdar W, Johannesson M, Yalcin B, Taylor MS, Taylor JM, Mott R, Flint J (2009). High resolution mapping of expression QTLs in heterogeneous stock mice in multiple tissues. Genome Res.

[40] Simonis M, Atanur SS, Linsen S, Guryev V, Ruzius FP, Game L, Lansu N, de Bruijn E, van Heesch S, Jones SJ, Pravenec M, Aitman TJ, Cuppen E (2012). Genetic basis of transcriptome differences between the founder strains of the rat HXB/BXH recombinant inbred panel. Genome Biol.

[41] Odell TT, Tausche FG, Lindsley DL, OWEN RD (1957). The homotransplantation of functional erythropoietic elements in the rat following total-body irradiation. Ann N Y Acad Sci.

[42] Nordquist N, Olofsson P, Vingsbo-Lundberg C, Petterson U, Holmdahl R (2000). Complex genetic control in a rat model for rheumatoid arthritis. J Autoimmun.

[43] Marta M, Stridh P, Becanovic K, Gillett A, Ockinger J, Lorentzen JC, Jagodic M, Olsson T (2010). Multiple loci comprising immune-related genes regulate experimental neuroinflammation. Genes Immun.

[44] Becanovic K, Bäckdahl L, Wallström E, Aboul-Enein F, Lassmann H, Olsson T, Lorentzen JC (2003). Paradoxical effects of arthritis-regulating chromosome 4 regions on myelin oligodendrocyte glycoprotein-induced encephalomyelitis in congenic rats. Eur J Immunol.

[45] Rintisch C, Kelkka T, Norin U, Lorentzen JC, Olofsson P, Holmdahl R (2010). Finemapping of the arthritis QTL Pia7 reveals co-localization with Oia2 and the APLEC locus. Genes Immun.

[46] Lorentzen JC, Flornes L, Eklöw C, Bäckdahl L, Ribbhammar U, Guo JP, Smolnikova M, Dissen E, Seddighzadeh M, Brookes AJ, Alfredsson L, Klareskog L, Padyukov L, Fossum S (2007). Association of arthritis with a gene complex encoding C-type lectin-like receptors. Arthritis Rheum.

[47] Harnesk K, Swanberg M, Ockinger J, Diez M, Lidman O, Wallström E, Lobell A, Olsson T, Piehl F (2008). Vra4 congenic rats with allelic differences in the class II transactivator gene display altered susceptibility to experimental autoimmune encephalomyelitis. J Immunol.

[48] Swanberg M, Lidman O, Padyukov L, Eriksson P, Akesson E, Jagodic M, Lobell A, Khademi M, Börjesson O, Lindgren CM, Lundman P, Brookes AJ, Kere J, Luthman H, Alfredsson L, Hillert J, Klareskog L, Hamsten A, Piehl F, Olsson T (2005). MHC2TA is associated with differential MHC molecule expression and susceptibility to rheumatoid arthritis, multiple sclerosis and myocardial infarction. Nat Genet.

[49] Ockinger JJ, Stridh PP, Beyeen ADA, Lundmark FF, Seddighzadeh MM, Oturai AA, Sørensen PSP, Lorentzen ARA, Celius EGE, Leppä VV, Koivisto KK, Tienari PJP, Alfredsson LL, Padyukov LL, Hillert JJ, Kockum II, Jagodic MM, Olsson TT (2010). Genetic variants of CC chemokine genes in experimental autoimmune encephalomyelitis, multiple sclerosis and rheumatoid arthritis. Genes Immun.

[50] Holm BC, Xu HW, Jacobsson L, Larsson A, Luthman H, Lorentzen JC (2001). Rats made congenic for Oia3 on chromosome 10 become susceptible to squalene-induced arthritis. Hum Mol Genet.

[51] Rintisch C, Ameri J, Olofsson P, Luthman H, Holmdahl R (2008). Positional cloning of the Igl genes controlling rheumatoid factor production and allergic bronchitis in rats. Proc Natl Acad Sci U S A.

[52] Olofsson P, Holmberg J, Tordsson J, Lu S, Akerström B, Holmdahl R (2003). Positional identification of Ncf1 as a gene that regulates arthritis severity in rats. Nat Genet.

[53] Wester L, Olofsson P, Ibrahim SM, Holmdahl R (2003). Chronicity of pristane-induced arthritis in rats is controlled by genes on chromosome 14. J Autoimmun.

[54] Olofsson P, Wernhoff P, Holmberg J, Holmdahl R (2003). Two-loci interaction confirms arthritis-regulating quantitative trait locus on rat chromosome 6. Genomics.

[55] Roth MP, Viratelle C, Dolbois L, Delverdier M, Borot N, Pelletier L, Druet P, Clanet M, Coppin H (1999). A genome-wide search identifies two susceptibility loci for experimental autoimmune encephalomyelitis on rat chromosomes 4 and 10. J Immunol.

[56] Baud A, Hermsen R, Guryev V, Stridh P, Graham D, McBride MW, Foroud T, Calderari S, Diez M, Ockinger J, Beyeen AD, Gillett A, Abdelmagid N, Guerreiro-Cacais AO, Jagodic M, Tuncel J, Norin U, Beattie E, Huynh N, Miller WH, Koller DL, Alam I, Falak S, Osborne-Pellegrin M, Martinez-Membrives E, Canete T, Blazquez G, Vicens-Costa E, Mont-Cardona C, Diaz-Moran S, Rat Genome Sequencing and Mapping Consortium (2013). Combined sequence-based and genetic mapping analysis of complex traits in outbred rats. Nat Genet.

[57] Blankenhorn EP, Descipio C, Rodemich L, Cort L, Leif JH, Greiner DL, Mordes JP (2007). Refinement of the Iddm4 diabetes susceptibility locus reveals TCRVbeta4 as a candidate gene. Ann N Y Acad Sci.

[58] Mattapallil MJ, Sahin A, Silver PB, Sun S-H, Chan C-C, Remmers EF, Hejtmancik JF, Caspi RR (2008). Common genetic determinants of uveitis shared with other autoimmune disorders. J Immunol.

[59] Guo JP, Bäckdahl L, Marta M, Mathsson L, Rönnelid J, Lorentzen JC (2008). Profound and paradoxical impact on arthritis and autoimmunity of the rat antigen-presenting lectin-like receptor complex. Arthritis Rheum.

[60] Brenner M, Meng H-C, Yarlett NC, Joe B, Griffiths MM, Remmers EF, Wilder RL, Gulko PS (2005). The non-MHC quantitative trait locus Cia5 contains three major arthritis genes that differentially regulate disease severity, pannus formation, and joint damage in collagen- and pristane-induced arth*ritis*. J Immunol.

[61] Hultqvist M, Sareila O, Vilhardt F, Norin U, Olsson LM, Olofsson P, Hellman U, Holmdahl R (2011). Positioning of a polymorphic quantitative trait nucleotide in the Ncf1 gene controlling oxidative burst response and arthritis severity in rats. Antioxid Redox Signal.

[62] Sawcer S, Hellenthal G, Pirinen M, Spencer CCA, Patsopoulos NA, Moutsianas L, Dilthey A, Su Z, Freeman C, Hunt SE, Edkins S, Gray E, Booth DR, Potter SC, Goris A, Band G, Bang Oturai A, Strange A, Saarela J, Bellenguez C, Fontaine B, Gillman M, Hemmer B, Gwilliam R, Zipp F, Jayakumar A, Martin R, Leslie S, Hawkins S, Giannoulatou E, International Multiple Sclerosis Genetics Consortium, Wellcome Trust Case Control Consortium 2 (2011). Genetic risk and a primary role for cell-mediated immune mechanisms in multiple sclerosis. Nature.

[63] Li H, Handsaker B, Wysoker A, Fennell T, Ruan J, Homer N, Marth G, Abecasis G, Durbin R, and 1000 Genome Project Data Processing Subgroup (2009). The Sequence alignment/map (SAM) format and SAMtools. Bioinformatics.

[64] McLaren W, Pritchard B, Rios D, Chen Y, Flicek P, Cunningham F (2010). Deriving the consequences of genomic variants with the Ensembl API and SNP Effect Predictor. Bioinformatics.

